# Physics-informed graph neural networks for real-time prediction of wall shear stress in stenotic coronary arteries

**DOI:** 10.1038/s41598-026-47410-z

**Published:** 2026-04-06

**Authors:** Ting-Ting Luo, Li Yang, Jie Chen, Zi-Wen Wu, Jie Chang, Yan-Tao Zhang

**Affiliations:** 1 Department of Pharmacy, The Second Affiliated Hospital of Wannan Medical College, Wuhu, 241000 People’s Republic of China; 2 School of Medical Information, Wannan Medical College, Wuhu, 241000 People’s Republic of China; 3 School of Computer and Information, Anhui Normal University, Wuhu, 241000 People’s Republic of China

**Keywords:** Physics-informed graph neural network, Coronary artery stenosis, Wall shear stress, Cardiology, Computational biology and bioinformatics, Engineering, Mathematics and computing

## Abstract

Wall shear stress (WSS) is a key hemodynamic parameter associated with atherosclerotic plaque development in coronary arteries. In this study, we developed a physics-informed graph neural network (PI-GNN) for efficient prediction of WSS distributions on stenotic coronary surfaces. Leveraging 40 subject-specific geometries reconstructed from coronary CT angiography, we employed statistical shape modeling to generate a cohort of 1000 synthetic models encompassing systematic variations in stenosis morphology (concentric and eccentric lesions, round and oval cross-sections, single and dual stenoses). Full computational fluid dynamics (CFD) simulations were performed to obtain ground-truth WSS data, which were then mapped onto vessel-surface graphs to train the proposed PI-GNN. The PI-GNN outperformed U-Net (R = 0.85) and multilayer perceptron (R = 0.24) baselines, achieving superior global performance (MAE = 1.05 Pa, RMSE = 5.63 Pa, R = 0.94) while maintaining robust accuracy across all stenosis scenarios. Node-wise Bland–Altman analysis demonstrated negligible mean bias (|bias|< 2 Pa) and narrow 95% limits of agreement, indicating reliable local agreement with CFD, even in complex severe and dual-lesion cases. With inference times reduced to seconds, the proposed PI-GNN serves as a computationally efficient surrogate for real-time clinical decision support and large-scale coronary hemodynamic studies.

## Introduction

### Motivation

Coronary artery disease (CAD) remains the leading cause of cardiovascular mortality worldwide^1^. It is characterized by the accumulation of atherosclerotic plaques within the arterial wall, which compromises luminal patency and disrupts myocardial perfusion^2,3^. The progression of these stenotic lesions is governed not merely by systemic factors but by the local hemodynamic environment arising from complex vessel morphologies^4–6^. Among hemodynamic parameters, Wall Shear Stress (WSS) serves as a critical mechanobiological regulator^7,8^. High WSS at stenotic throats increases the risk of plaque rupture and subsequent thrombotic events, while Low WSS promotes plaque progression through inflammatory pathways^9,10^. Quantitatively, WSS values below approximately 1.0 Pa are considered pathologically low, promoting lipid accumulation and inflammatory cell adhesion through disturbed flow patterns, while regions of elevated WSS have been associated with plaque destabilization, fibrous cap thinning, and increased vulnerability to rupture through mechanical overstress and inflammatory activation^11^. Consequently, the accurate mapping of WSS distributions is indispensable for patient-specific risk stratification.

While computational fluid dynamics (CFD) represents the standard for non-invasive WSS quantification, its prohibitive computational cost and reliance on expert manual preprocessing render it unsuitable for real-time clinical decision-making. Moreover, the complex, tortuous geometry of coronary arteries poses a fundamental challenge to conventional deep learning surrogates (e.g., convolutional neural networks). Therefore, there is an imperative need for a computational paradigm that reconciles the high fidelity of physics-based simulations with the inference efficiency of data-driven models. Recent advancements in two complementary yet independent computational paradigms, namely graph neural networks (GNNs) for handling irregular geometric domains and physics-informed neural networks (PINNs) for incorporating governing physical laws, offer promising opportunities to overcome these computational challenges^12^.

### Literature review

The morphological characteristics of coronary artery stenotic lesions, such as shape, size, and severity, significantly influence local hemodynamics and are critical for assessing cardiovascular risk^13–16^. While intravascular imaging modalities like IVUS and OCT offer high-resolution anatomical data^17–19^, their invasive nature precludes their use in routine risk screening. Consequently, CFD derived from non-invasive coronary CT angiography (CCTA) has become the standard for functional assessment^20,21^. Extensive computational studies have confirmed that complex geometric features induce distinct flow disturbances and shear stress alterations that closely correlate with disease progression ^22–24^. However, despite its high fidelity, CFD remains computationally expensive, typically requiring hours of processing per patient, which creates a bottleneck for large-scale clinical deployment.

To circumvent these computational constraints, machine learning (ML) approaches have emerged as compelling alternatives for hemodynamic prediction. Early applications primarily utilized convolutional neural networks (CNNs), such as U-Net architectures, to capture geometry-hemodynamics relationships. Pioneering work by Su et al. demonstrated that CNN-based models could predict WSS in idealized coronary arteries with a 2.5% normalized mean absolute error, reducing computation time from hours to seconds^24^. However, these purely data-driven approaches face intrinsic limitations regarding generalization, particularly when applied to complex patient-specific anatomies. This limitation stems largely from the requirement of CNNs to project irregular vascular geometries onto structured grids (voxelization), which often leads to a loss of surface topology and spatial fidelity. An alternative strategy for accelerating hemodynamic computation is reduced-order modeling (ROM), including one-dimensional simplifications of the Navier–Stokes equations and projection-based methods such as POD-Galerkin models. These approaches offer extremely fast inference without requiring training data and generalize naturally to parametric variations^25,26^. However, 1D ROM approaches produce axially-averaged hemodynamic quantities and cannot resolve the circumferential WSS variations that are clinically important in eccentric or asymmetric stenoses, while POD-Galerkin methods require a pre-computed snapshot basis from full CFD simulations for each new parametric configuration.

A transformative advancement emerged with the introduction of physics-informed neural network (PINN)^27^, which embed governing physical laws (e.g., Navier–Stokes equations) directly into the loss function to ensure physically consistent predictions. Building on this, PI-GNN has evolved to combine physical rigor with geometric flexibility, leveraging graph structures to represent vascular domains without the need for structured remeshing. Gao et al. demonstrated PI-GNN’s capability in predicting flow fields within irregular domains, achieving CFD-level accuracy with orders of magnitude in computational speedup^28^. In cardiovascular applications, Arzani et al. successfully applied physics-informed approaches to capture WSS patterns in patient-specific aortic aneurysms^29^. The synergy between graph-based geometric representation and physics-informed constraints makes PI-GNNs particularly suited for coronary arteries with their tortuous paths and variable cross-sections. Despite these advances, the specific application of PI-GNNs to coronary stenosis WSS prediction remains largely unexplored, presenting a unique opportunity to combine geometric flexibility with physical rigor for real-time WSS prediction.

To bridge these gaps, this study presents a novel framework that synergizes physics-informed graph learning with statistical data augmentation. Unlike previous works limited by data scarcity, we leveraged statistical shape modeling (SSM) to extract morphological priors from 40 subject-specific coronary geometries, expanding them into a massive, physiologically plausible synthetic cohort of 1000 models. This strategy ensures that our proposed PI-GNN is trained on a dataset that encompasses a systematic and comprehensive variation of stenosis morphologies (including concentric, eccentric, and serial lesions), thereby achieving a balance between the high fidelity of physics-based simulations and the real-time efficiency required for clinical decision support.

### Paper organization

The rest of this paper is organized as follows. Section “Methodology” describes the methodology, including coronary artery geometry generation, CFD simulation setup, and the implementation of three machine learning models. Section “Results and discussion” evaluates model performance across different stenosis severities and morphologies, comparing prediction accuracy. Finally, Section “Discussion” presents the conclusion and outlook.

## Methodology

The methodology for developing the WSS prediction framework consists of three sequential components: (i) Coronary artery geometry modeling; (ii) CFD simulation setup; and (iii) Machine learning architectures. The specific details are as follows.

### Coronary artery geometry modeling

To ensure anatomical fidelity and physiological relevance, the baseline geometric features were derived from clinical coronary CT Angiography (CCTA) data of 40 subjects (20 healthy controls, 20 with atherosclerosis)^30^. The dataset comprised 96 left coronary artery (LCA) branches and 65 right coronary artery (RCA) branches. Preprocessing included surface smoothing, distal clipping, centerline extraction using the Vascular Modeling Toolkit (VMTK), which computes paths of maximal inscribed sphere centers connecting inlet and outlet seed points to identify the geometric medial axis and local lumen radius at each centerline point; extracted centerlines were uniformly resampled to 640 points along the arc-length coordinate, and remeshing, to obtain high-quality lumen surfaces suitable for hemodynamic simulations and shape analysis. For each branch, the extracted centerline was parameterized by arc length $s$ and represented as a sequence of 3D coordinates $r(s) = [(x(s),y(s),z(s)]$ together with the local lumen radius $R(s)$. Basic geometric descriptors were then computed to characterize vessel length and tortuosity. The path length was defined as

1 $$l = s_{\max } - s_{\min } ,$$

and the chord length as

2 $$c = ||r(s_{end} ) - r(s_{start} )||.$$

From these, the sinuosity $S = l/c$ was calculated, which indicates how tortuous or meandering the vessel path is (with $S = 1$ corresponding to a straight vessel and larger values indicating more bending). In addition, the local curvature $\kappa (s)$ (unit: mm⁻^−1^) was evaluated to quantify the intensity of bending along the centerline, using the Frenet–Serret relation

3 $$\kappa (s) = \frac{||r^{\prime}(s) \times r^{\prime\prime}(s)||}{{||r^{\prime}(s)||^{3} }}.$$

The torsion $\tau (s)$ (unit: mm^−1^), which characterizes the three-dimensional twisting of the vessel path, is given by

4 $$\tau (s) = \frac{\det (r\prime (s),r\prime \prime (s),r\prime \prime \prime (s))}{{||r\prime (s) \times r\prime \prime (s)||^{2} }},$$

where $r\prime (s)$,$r\prime \prime (s)$ and $r\prime \prime \prime (s)$ are the first, second, and third derivatives of the centerline position vector with respect to the arc-length parameter $s$ (mm), respectively..

Building on these real-world measurements, we adopted a statistical shape modeling strategy to generate a large cohort of anatomically plausible but diverse coronary centerlines. For each real branch, we computed a curvature-based descriptor vector (including sinuosity and several integral measures of curvature) and estimated its empirical joint distribution separately for LCA and RCA branches. New synthetic centerlines were then obtained by randomly selecting a real branch and applying a trajectory-level diffeomorphic deformation based on large deformation diffeomorphic metric mapping (LDDMM), with the deformation constrained so that the resulting descriptor vector remained within these empirical distributions^31^. Gaussian smoothing of the deformation momenta was used to promote smooth path transitions rather than local noise. After deformation, each synthetic centerline was rescaled to 80 mm and uniformly resampled to 640 points, providing a standardized representation for subsequent mesh generation and model training.

Each synthetic model incorporated 1–2 focal stenoses with randomized orientation and axial location to extend beyond axisymmetric cases. A normal diameter $D_{0} = 3$ mm was adopted, and stenotic area ratio $AR \in [0.1,0.9]$ was prescribed as

5 $$AR = \frac{{A_{sten} }}{{A_{0} }},$$

where $A_{sten}$ and $A_{0}$ denote the stenotic cross-sectional and normal areas, respectively.

**Fig. 1:**
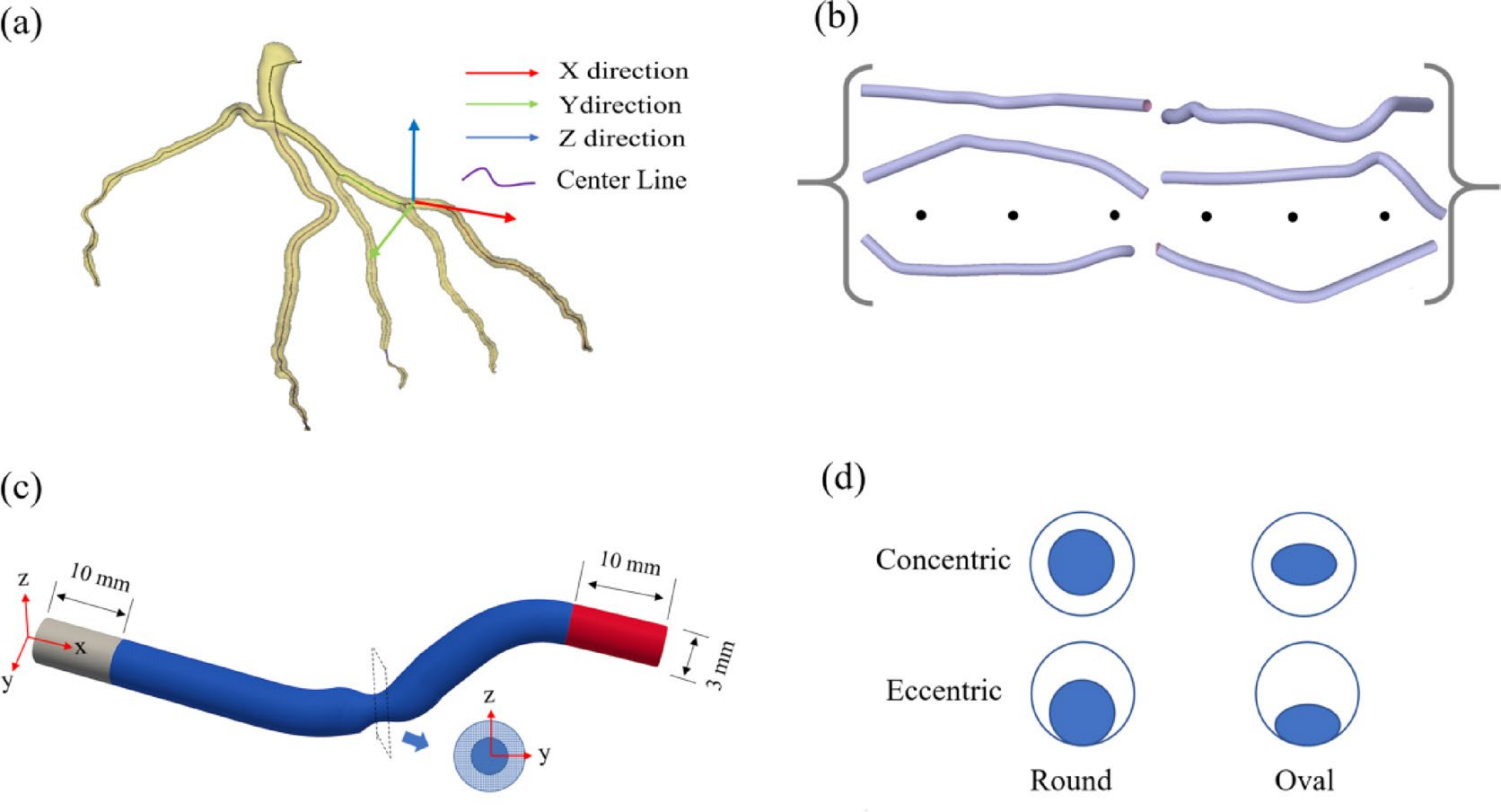
Schematic diagram of coronary artery modelling with different stenosis shapes. (**a**) Coronary artery tree; (**b**) Reconstructed 3D coronary artery model; (**c**) Coronary artery stenosis model; (**d**) Cross-sectional shapes of the stenosis.

Four morphology classes were implemented—concentric circular, eccentric circular, concentric elliptical, and eccentric elliptical—with optional combinations. For eccentric cases, the throat center was offset within the cross-sectional plane by as much as 50% of the local radius. Stenoses were randomly positioned between 10–70 mm from the inlet. Each model used 640 centerline samples and 40 circumferential angles, producing VTP centerlines, STL surfaces, and CSV coordinate data. Figure 1a–d illustrate the coronary artery tree and its centerline, the reconstructed three-dimensional coronary artery model, the coronary artery stenosis model, and four types of stenosis morphology.

### CFD simulation setup

A systematic mesh-generation pipeline was developed to balance numerical accuracy and computational efficiency. The workflow began with converting STL surface meshes into STP solid geometry models to reconstruct the triangulated surface as continuous spline patches, which effectively repairs potential geometric defects inherent in STL tessellations, such as non-manifold edges, gaps between adjacent facets, degenerate triangles, and self-intersections that could otherwise compromise mesh quality and numerical stability during CFD simulation. A hybrid meshing strategy was implemented, strategically combining structured hexahedral boundary layers near vessel walls for precise WSS capture with polyhedral core meshes for enhanced geometric adaptability. Grid-independence analysis was conducted across two orders of magnitude, with element sizes ranging from 1.0 mm to 0.01 mm, corresponding to total mesh counts of 50,000–800,000 elements. WSS convergence was evaluated using a concentric circular stenosis model with a post-stenotic diameter of 1.2 mm, corresponding to a 16% stenotic area ratio relative to the normal lumen. A steady inlet velocity of 0.2 m/s, representing the cycle-averaged coronary flow velocity used in the transient simulations, and zero gauge pressure at the outlet were imposed. At the stenotic throat (the narrowest cross-section of the concentric circular stenosis test model), the solution stabilized at 92.3 Pa using 0.05 mm elements (approximately 300,000 total elements). This configuration achieved a 60% reduction in computational cost compared with the finest mesh while maintaining WSS accuracy within 1%. All mesh elements satisfied stringent quality criteria, with orthogonality exceeding 0.85 and aspect ratios below 10:1, ensuring numerical stability for high-throughput simulations.

Hemodynamic simulations were performed using the ANSYS Fluent solver (ANSYS, Inc., Canonsburg, PA, USA), a finite-volume-based commercial CFD platform that has been extensively employed for cardiovascular hemodynamic simulations in the literature^15,32,33^. Here we utilized the PyFluent API for automated batch processing to enable parallel computing. A second-order implicit temporal discretization scheme captured the transient flow characteristics, with laminar flow assumptions appropriate for coronary Reynolds numbers (Re≈500). The finite volume method discretized the incompressible Navier–Stokes and continuity equations:

6 $$\frac{\partial u}{{\partial t}} + \rho (u \cdot \nabla )u = - \nabla p + \mu \nabla^{2} u,$$

7 $$\nabla \cdot u = 0,$$

where $u$ refers to velocity vector, $\rho$ represents blood density, $\nabla$ is the spatial gradient operator, $p$ means pressure, $\mu$ denotes the dynamic viscosity of blood and $\nabla^{2}$ is the Laplacian operator.

**Fig. 2:**
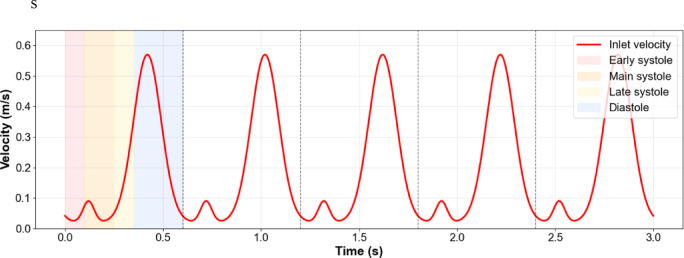
Physiological coronary inlet velocity waveform used as boundary condition.

Simulations employed a time step of 1 × 10⁻^5^ s over five cardiac cycles (3.0 s total), ensuring fully developed flow fields and elimination of initial transient effects. Rigid vessel walls were assumed with no-slip boundary conditions. At the proximal inlet of each coronary side branch, a physiologically motivated, diastole-dominant velocity waveform $u{}_{in}$ was prescribed (Fig. 2). The waveform consists of a small systolic shoulder and a dominant diastolic peak, with a peak velocity of approximately 0.55 m/s and a cycle-averaged velocity of 0.20 m/s, consistent with reported coronary flow measurements in the proximal epicardial arteries^34^. At the distal outlet, a constant pressure boundary condition of 100 mmHg (≈ 13.3 kPa) was applied, representing the mean aortic pressure between a diastolic value of ≈ 80 mmHg and a systolic peak of ≈ 120 mmHg^31^. Convergence criteria of 1 × 10⁻⁶ were enforced for both continuity and momentum residuals. Blood rheology was modeled using the Carreau non-Newtonian law to account for shear-thinning behavior at the low to moderate shear rates typical of coronary artery flow. Wall shear stress (WSS), the primary hemodynamic quantity of interest for atherosclerosis assessment, was extracted from each simulation for subsequent machine-learning model training.

### Machine learning models

The prediction of coronary artery WSS represents a supervised regression task that maps continuous geometric inputs to continuous hemodynamic outputs. Given the complex nonlinear relationships between vessel morphology and flow patterns, we considered three representative deep-learning architectures with increasing model complexity and physical awareness: a Multi-Layer Perceptron (MLP), a U-Net and a Physics-Informed Graph Neural Network (PI-GNN). The MLP serves as a baseline deep-learning approach but, when applied to the full high-dimensional data, would be computationally prohibitive, necessitating dimensionality-reduction techniques such as autoencoder-based compression before fully connected layers are used for global feature mapping. The U-Net, a CNN-based architecture, naturally addresses spatial correlations through convolutional operations and skip connections that preserve both local geometric details and global vessel context. However, because such purely data-driven models do not explicitly encode the governing flow physics, their generalization to complex patient-specific stenoses remains uncertain. To address this limitation, we develop PI-GNN, which combines graph-based geometric representation with physics-informed constraints derived from the Navier–Stokes equations, thereby aiming to improve accuracy and robustness for complex stenotic geometries.

#### Input and output variables

**Fig. 3:**
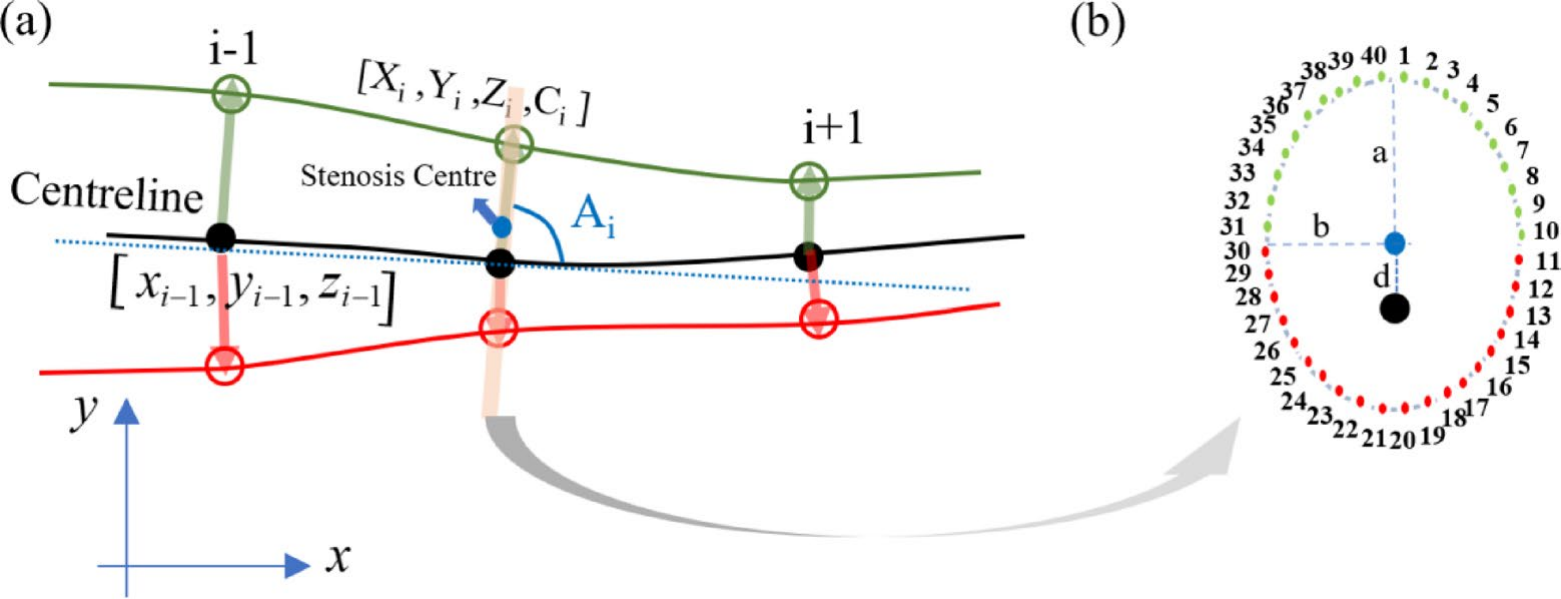
(**a**) Surface coordinates (X, Y, Z), curvature (C), and angle (A) are used as input features to represent coronary artery geometry. (**b**) Geometric parameters of oval stenosis: a and b denote the semi-major and semi-minor axes, and d is the offset from the centerline.

In this study, geometric features of coronary arteries serve as input variables, while the wall shear stress (WSS) magnitude distributions constitute the output, aiming to replace computationally expensive CFD simulations for real-time clinical applications. As shown in Fig. 3, the input features comprise an 8-dimensional vector of geometric descriptors extracted from each vessel model. These include coronary artery surface coordinates (X, Y, Z), the discretized curvature magnitude C derived from the continuous curvature $\kappa (s)$, the local angle A, and stenosis-specific parameters: the eccentric distance from stenosis center to centerline d, and the semi-major a and minor b axis lengths of the stenosis cross-section. The local angle A represents the circumferential position of each surface node within the cross-sectional plane perpendicular to the centerline at axial station i (Fig. 3a), defined as A = 2π(k-1)/40 for the k-th node (k = 1, 2, …, 40, as numbered in Fig. 3b), which enables the network to capture non-uniform circumferential WSS distributions in eccentric and oval stenoses. All synthetic geometries are aligned to a canonical coordinate system with the inlet centered at the origin and the inlet normal along the positive z-axis. To prevent boundary effects and ensure stable flow development, we exclude 5 mm transition zones at both inlet and outlet regions, resulting in an effective analysis length of 70 mm. These features are sampled at 560 axial positions (spaced at 0.125 mm intervals) and 40 circumferential points, yielding a structured input tensor of dimensions 560 × 40 × 8.

Critically, all 40 circumferential points must be retained for each axial position to preserve the geometric complexity of elliptical and eccentric stenoses. Dimensional reduction would result in the loss of essential asymmetric features that characterize irregular plaque distributions commonly observed in clinical cases. This full circumferential representation ensures accurate capture of non-axisymmetric geometries and their associated flow disturbances. For the output variable, WSS values from CFD simulations undergo spatial interpolation to ensure consistency with the input geometry structure. While WSS is inherently a three-dimensional vector quantity, we focus on its magnitude, which is the primary hemodynamic parameter associated with atherosclerosis development. The irregular CFD mesh outputs are mapped to the corresponding 560 × 40 grid points using nearest-neighbor interpolation, creating a standardized WSS magnitude matrix. This regularization strategy transforms unstructured CFD results into structured datasets suitable for neural network training. The resulting paired input–output datasets establish a direct mapping between geometric features and hemodynamic responses, providing the foundation for training machine learning models capable of predicting WSS distributions directly from coronary geometry. This approach enables rapid hemodynamic assessment without requiring CFD computations.

#### Multi-layer perceptron (MLP)

The direct application of a multi-layer perceptron (MLP) to coronary WSS prediction presents substantial computational challenges due to the high dimensionality of both input and output data. Specifically, the input comprises 560 axial points, 40 circumferential points, and 8 geometric features, resulting in 179,200 input features, while the output consists of 560 × 40 = 22,400 WSS values. A fully connected network processing this scale of data would require over 4.0 billion parameters in the first layer alone, leading to significant computational inefficiency, including excessive memory usage and extended training time. Moreover, the curse of dimensionality introduces a high risk of overfitting, especially given the limited availability of training samples relative to the parameter space.

**Fig. 4:**
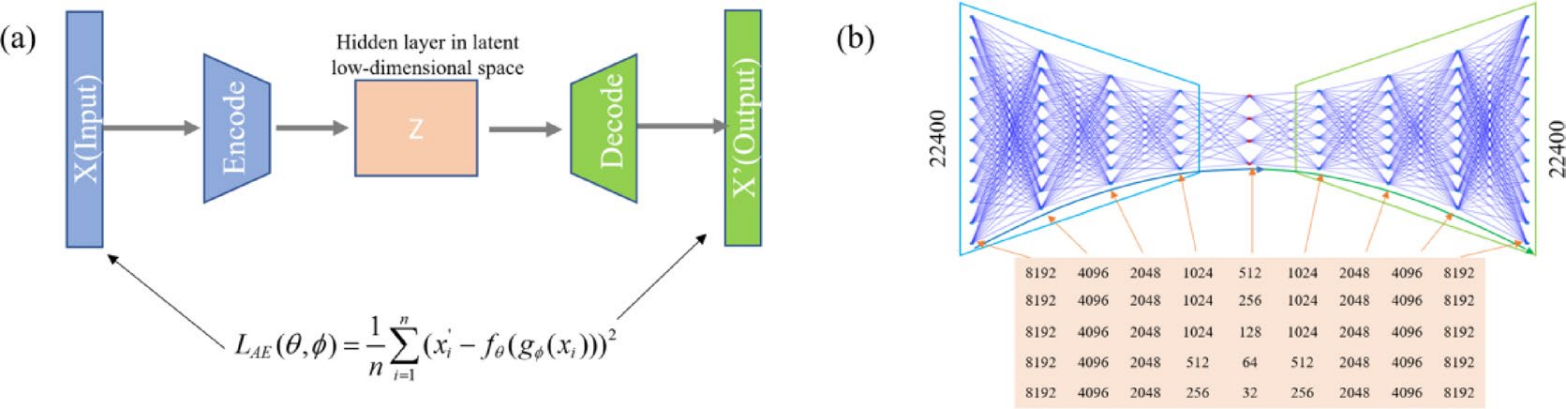
Schematic view of autoencoder architecture. (**a**) Encoder–decoder framework, where X (input) is compressed into latent space Z and reconstructed as X’(Output); (**b**) MLP-based autoencoder structure with symmetric layer configuration.

To overcome these limitations, this study adopts a dual autoencoder-based dimensionality reduction approach that compresses both the high-dimensional input features and output WSS values into compact latent representations while preserving essential information for accurate mapping. As illustrated in Fig. 4, the framework consists of three sequential stages: (1) input compression via convolutional autoencoder that captures spatial correlations while reducing the geometric features from 179,200 to a compact latent dimension, (2) latent space mapping via a compact MLP network with two hidden layers (100 neurons each) employing ReLU activation functions and dropout regularization (rate = 0.2), and (3) output reconstruction via decoder that transforms the predicted latent representation back to the full WSS field. Multiple candidate bottleneck dimensions (32, 64, 128, 256, and 512) are evaluated to determine the optimal trade-off between compression ratio and reconstruction accuracy, with the autoencoder framework employing the reconstruction loss function

8 $$L_{AE} (\theta ,\phi ) = \frac{1}{n}\sum\limits_{i = 1}^{n} {(x_{i}^{\prime } - f_{\theta } (g_{\phi } (x_{i} )))^{2} } ,$$

where $g_{\phi }$ and $f_{\theta }$ denote the encoder and decoder functions, respectively.

**Fig. 5:**
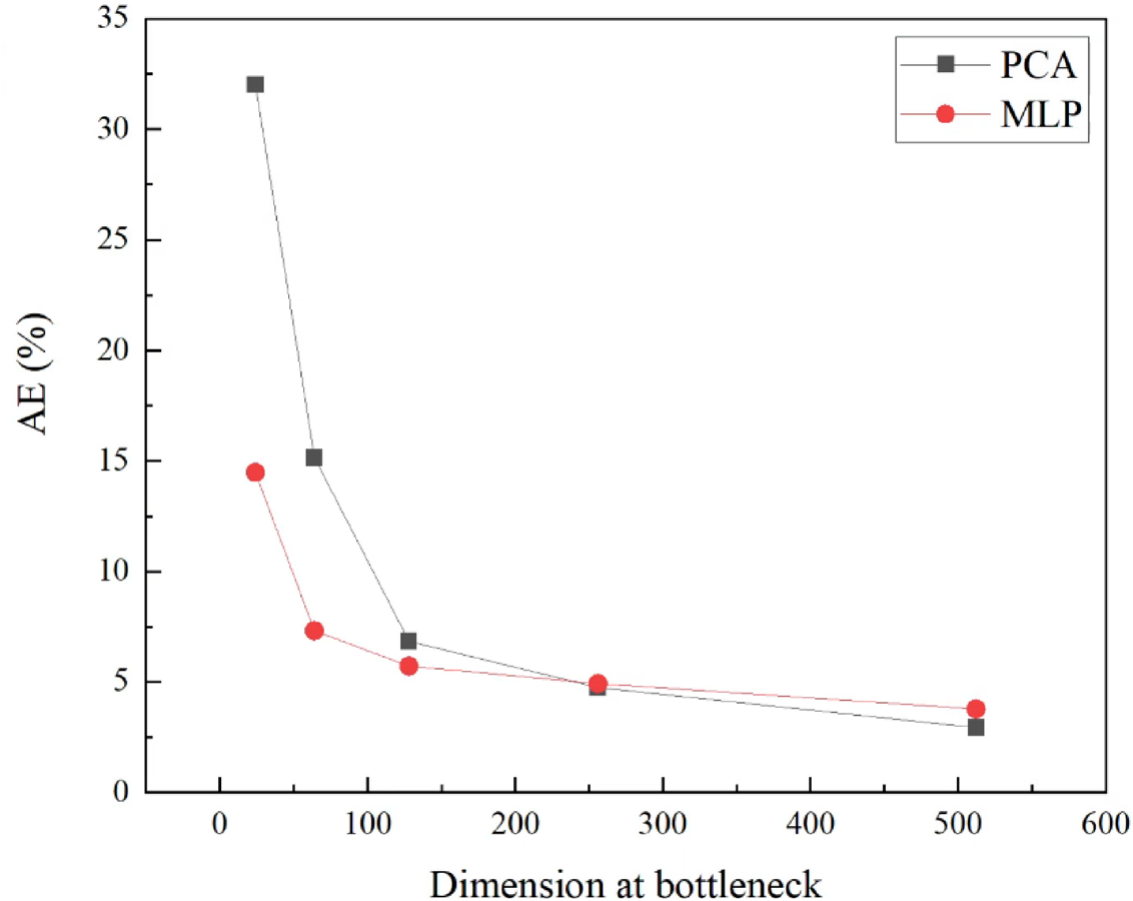
NMAE under different bottleneck dimension. PCA is considered as the baseline.

We systematically compared two dimensionality reduction techniques to determine the optimal bottleneck dimension: Principal Component Analysis (PCA) and Multi-Layer Perceptron Autoencoder. Five candidate bottleneck dimensions (32, 64, 128, 256, and 512) were evaluated, with reconstruction accuracy quantified using the Mean Absolute Error (MAE) between original and reconstructed WSS data. As illustrated in Fig. 5, both methods demonstrated decreasing reconstruction errors with increasing dimensionality. PCA exhibited MAE values decreasing from 32.0% at 32 dimensions to 2.7% at 512 dimensions, indicating that the WSS fields in the current dataset are dominated by smooth, large-scale spatial structures that are well captured by PCA’s linear basis representation. The MLP-based autoencoder demonstrated superior performance with MAE decreasing from 14.5% at 32 dimensions to 3.6% at 512 dimensions, benefiting from its ability to capture nonlinear relationships in the data. Considering the trade-off between reconstruction fidelity and computational efficiency, a bottleneck dimension of 128 was selected, where MLP-based autoencoder achieved 5.8% MAE compared to 6.8% for PCA. This dual compression strategy enables the MLP to learn mappings between compressed representations rather than original high-dimensional spaces, substantially reducing computational burden from billions to millions of parameters while preserving essential nonlinear relationships for accurate real-time WSS prediction.

#### U-Net architecture

**Fig. 6:**
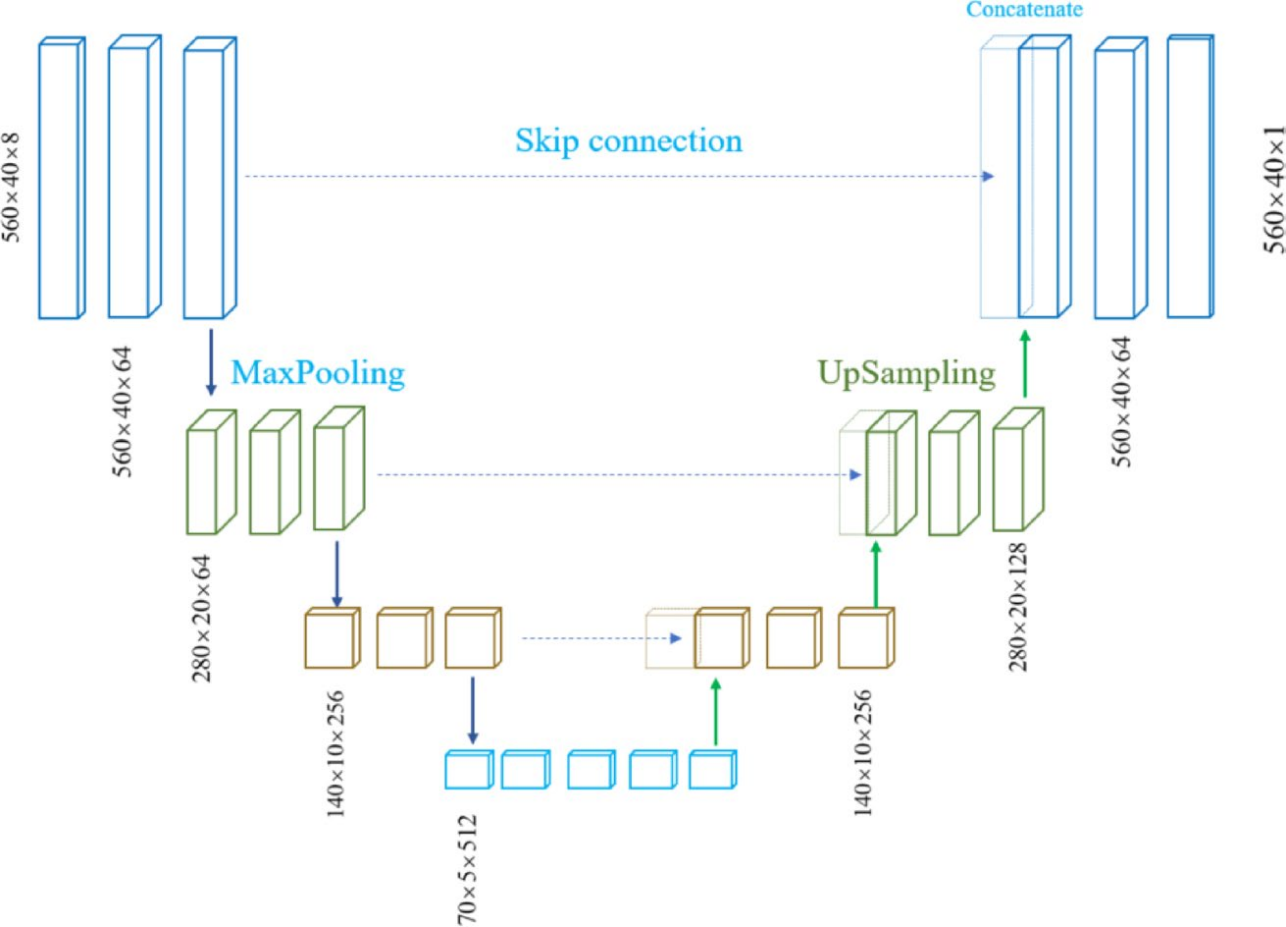
U-Net architecture with skip connections for WSS prediction. Input size is 560 × 40 × 8, featuring a symmetric encoder–decoder design and a 512-channel bottleneck.

The U-Net architecture employed in this study directly processes the high-dimensional input data (560 × 40 × 8) without requiring explicit dimensionality reduction through autoencoders. As illustrated in Fig. 6, this end-to-end learning approach leverages the inherent capability of convolutional operations to preserve spatial relationships while extracting hierarchical features, making it particularly suitable for the structured nature of coronary WSS data. Originally developed for biomedical image segmentation, U-Net’s distinctive symmetric encoder-decoder structure with skip connections proves highly effective for regression tasks involving spatially structured data.

The encoder path implements a systematic feature extraction strategy through convolutional blocks, progressively increasing feature channels (64 → 128 → 256) while reducing spatial dimensions via 2 × 2 max pooling operations. Each block contains two 3 × 3 convolutional layers followed by batch normalization and ReLU activation, ensuring stable gradient flow during training. The bottleneck layer processes the most abstract representations with 512 filters, serving as the semantic core of the model. The decoder path symmetrically reconstructs spatial resolution through transpose convolution upsampling operations, halving feature channels at each level (256 → 128 → 64). The critical innovation lies in the skip connections that concatenate corresponding encoder feature maps with decoder outputs, preserving fine-grained spatial information that would otherwise be lost during down-sampling. This architecture effectively combines low-level geometric details with high-level semantic features.

To optimize training stability and generalization, the network incorporates several regularization techniques. Dropout rates increases from 0.1 to 0.3 with network depth, and batch normalization is applied after each convolutional layer. The model employs a combined loss function that integrates three complementary terms to ensure accurate WSS prediction while maintaining spatial coherence:

9 $$L_{U - NET} = L_{MSE} + \lambda_{1} L_{relative} + \lambda_{2} L_{smoothness} ,$$

where $L_{MSE} = \frac{1}{N}\sum\limits_{i = 1}^{N} {||y_{i} - \mathop y\limits^{ \wedge }_{i} ||^{2} }$ is the mean squared error, $L_{relative} = \frac{1}{N}\sum\limits_{i = 1}^{N} {\frac{{|y_{i} - \mathop y\limits^{ \wedge }_{i} |}}{{|y_{i} | + \varepsilon }}}$ ensures normalized performance across different WSS magnitudes, and the spatial smoothness term $L_{smoothness} = \frac{1}{N}\sum\limits_{i = 1}^{N} {[(\nabla_{x} y_{i} )^{2} + (\nabla_{y} \mathop y\limits^{ \wedge }_{i} )^{2} ]}$ promotes coherent WSS fields by penalizing abrupt spatial variations. The weighting parameters are set to $\lambda_{1} = 0.1$ and to $\lambda_{2} = 0.05$ balance the contribution of each loss component. The final 1 × 1 convolutional layer produces single-channel WSS predictions without activation function, appropriate for regression tasks.

This U-Net architecture achieves superior performance by eliminating the computational overhead of separate compression steps while maintaining the ability to learn complex nonlinear mappings between coronary geometry and WSS distribution. The end-to-end learning paradigm enables direct optimization for the WSS prediction task, potentially improving accuracy compared to multi-stage approaches. The integrated loss formulation ensures that the model not only minimizes prediction errors but also maintains physically meaningful spatial coherence in the predicted WSS fields.

#### Physics-informed graph neural network (PI-GNN)

While purely data-driven approaches like MLP and U-Net have shown promise in WSS prediction, they inherently lack the physical constraints that govern blood flow dynamics, particularly in capturing the complex wake patterns downstream of stenotic regions. To address this limitation, we develop a Physics-Informed Graph Neural Network (PI-GNN) that integrates fundamental fluid dynamics principles with graph-based learning architectures. The framework incorporates physically derived soft constraints based on analytical relationships between WSS and local vessel geometry into the loss function, thereby guiding the network toward hemodynamically plausible predictions^12^. This novel approach represents coronary arteries as graph structures where nodes correspond to the 560 × 40 surface mesh points and edges encode spatial relationships based on both grid connectivity and geometric proximity, enabling natural handling of complex vascular geometries while enforcing physical consistency in regions with significant flow disturbances.

**Fig. 7:**
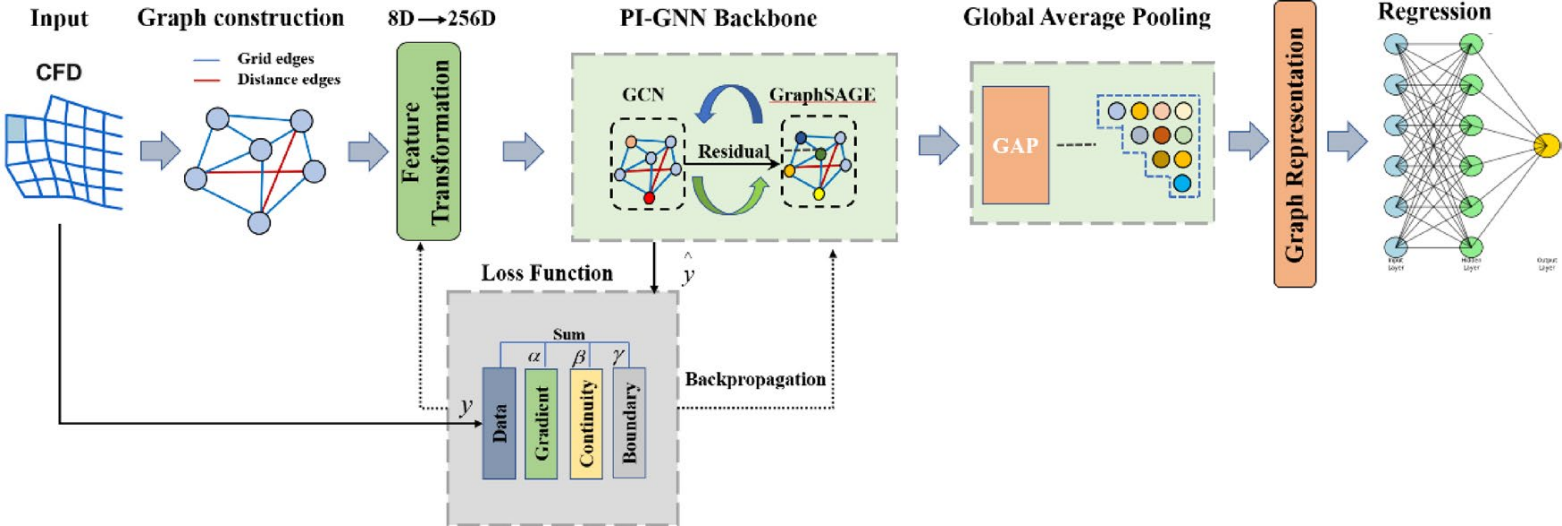
Schematic diagram of PI-GNN structure.

As illustrated in Fig. 7 the PI-GNN architecture consists of four key components: (1) a graph construction module that transforms the structured input tensor into a graph representation with hybrid edge connectivity, (2) an input transformation layer that projects the 8-dimensional geometric features to a 256-dimensional hidden space, (3) alternating GCN and GraphSAGE layers with residual connections for multi-scale feature extraction, and (4) a physics-informed loss function that incorporates hemodynamic constraints through multiple constraint terms.

For GCN layers (even indices):

10 $$h_{i}^{(l + 1)} = \sigma (\sum\limits_{{j \in N(i)U\{ i\} }} {\frac{1}{{\sqrt {\mathop {d_{i} }\limits^{ \wedge } \mathop d\limits^{ \wedge }_{j} } }}} h_{j}^{(l)} )W^{(l)} .$$

For GraphSAGE layers (odd indices):

11 $$h_{i}^{(l + 1)} = \sigma (W_{1}^{(l)} h_{i}^{(l)} + W_{2}^{(l)} MEAN_{j \in N(i)} h_{j}^{(l)} ),$$

where $h_{i}^{(l)}$ represents the feature vector of node $i$ at layer $l$, $N(i)$ denotes the neighborhood of node $i$, $\mathop {d_{i} }\limits^{ \wedge }$ is the degree of node $i$ with added self-loops, and $W_{{}}^{(l)}$, $W_{1}^{(l)}$ $W_{2}^{(l)}$ are learnable weight matrices. The alternating architecture leverages GCN’s spectral approach for smooth feature propagation and GraphSAGE’s sampling-based aggregation for capturing local variations.

The graph construction employs a hybrid connectivity strategy that combines structured grid neighbors with distance-based connections:

12 $$\varepsilon = \varepsilon_{grid} \cup \varepsilon_{spatial} ,$$

where $\varepsilon_{grid}$ denotes the set of edges connecting each node to its 8-connected neighbors in the structured 560 × 40 grid (4 direct axial/circumferential neighbors and 4 diagonal neighbors). $\varepsilon_{spatial}$ means additional edges connecting nodes within a spatial proximity threshold based on Euclidean distance in 3D space, whose edge weights are computed as:

13 $$w_{ij} = \exp ( - \frac{{||x_{i} - x_{j} ||}}{0.1}).$$

where $||x_{i} - x_{j} ||$ is the Euclidean distance between nodes $i$ and $j$, and the length scale parameter 0.1 is chosen to match the characteristic mesh spacing, ensuring rapid weight decay beyond immediate spatial neighbors. This weighting scheme ensures stronger influence from spatially proximate nodes while maintaining global connectivity. The critical innovation of PI-GNN lies in its physics-informed loss function, which combines data-driven WSS prediction error with physical constraint violations:

14 $$L_{PI - GNN} = L_{MSE} + \alpha L_{gradient} + \beta L_{continuity} + \gamma L_{boundary} ,$$

where $L_{MSE} = \frac{1}{N}\sum\limits_{i = 1}^{N} {||y_{i} - \mathop y\limits^{ \wedge }_{i} ||^{2} }$ is the mean squared error between predicted and CFD-derived WSS values; $L_{gradient} = \frac{1}{\varepsilon }\sum\nolimits_{(i,j) \in \varepsilon } {||\nabla_{ij} \mathop y\limits^{ \wedge } - } \nabla_{ij} \mathop y\limits^{{}} ||^{2}$ enforces consistency of spatial WSS gradients between neighboring nodes, where $\varepsilon$ denotes the edge set and $\nabla_{ij}$ represents the finite-difference gradient along edge $(i,j)$; $L_{continuity} = \frac{1}{N}\sum\nolimits_{i = 1}^{N} {\left\| {\frac{{d\mathop {y_{i} }\limits^{ \wedge } }}{ds} + \mathop y\limits^{ \wedge }_{i} \cdot (\frac{{d(\ln R_{i} )}}{ds} + \kappa_{i} \cdot \cos \theta_{i} )} \right\|}^{2}$ is a continuity-inspired constraint that penalizes WSS variations inconsistent with local geometric changes, where $R_{i}$ (mm) is the local radius, $\kappa_{i}$ (mm⁻^1^) is the local curvature,$s$ (mm) is the arc-length coordinate, and $\theta_{i}$ (rad) is the angle between the flow direction and the curvature plane; and $L_{boundary} = \frac{1}{{N_{inlet} }}\sum\limits_{i = 1}^{{N_{inlet} }} {||\mathop {\tau_{i} }\limits^{ \wedge } - \tau_{inlet} ({\mathrm{Re}} ,\kappa )} ||^{2}$ physically reasonable inlet WSS conditions based on the analytical Poiseuille solution adjusted for local curvature effects, where Re is the Reynolds number and $\kappa$ is the centerline curvature at the inlet cross-section.

The model employs adaptive weight scheduling with $\alpha$ = 0.1, $\beta$ = 0.05, and $\gamma$ gradually increasing from 0.01 to 0.1 over 500 epochs, allowing the network to first learn the data distribution before enforcing physical constraints, ensuring stable convergence while maintaining the ability to capture complex wake patterns downstream of stenotic regions that are critical for accurate hemodynamic assessment.

#### Training configuration

The experimental framework was designed to ensure robust and fair comparison across all three proposed architectures (MLP, U-Net, and PI-GNN). The dataset comprising 1,000 geometric models was partitioned using a standard 80/10/10 split, providing 800 models for training, 100 for hyperparameter tuning and validation during training, and 100 for performance evaluation on unseen data. Data preprocessing was standardized across all models, involving logarithmic transformation of WSS values to manage their typically skewed distribution, followed by normalization to zero mean and unit variance to stabilize training processes and accelerate convergence. All models were trained using the Adam optimizer with an initial learning rate of 0.001, dynamic learning rate adjustment through ReduceLROnPlateau callback (factor = 0.5, patience = 10 epochs), and early stopping mechanism (patience = 30 epochs) to prevent overfitting and ensure optimal generalization capability.

Each architecture employed tailored training configurations optimized for their respective characteristics. The MLP framework utilized a two-stage training process: first training the dual autoencoders for dimensionality reduction with reconstruction loss, then training the compact MLP network for latent space mapping. The U-Net architecture employed end-to-end training with a composite loss function integrating mean squared error, relative error, and spatial smoothness terms. The PI-GNN incorporated a curriculum learning strategy with adaptive weight scheduling (α = 0.1, β = 0.05, $\gamma$ gradually increasing from 0.01 to 0.1 over the training period), allowing the network to first learn data distributions before enforcing physics-informed constraints. All models were trained for a maximum of 500 epochs with batch size of 4, utilizing appropriate regularization techniques including dropout layers and batch normalization.

## Results and discussion

This section presents a comprehensive comparative analysis of the three proposed machine learning architectures (MLP, U-Net, and PI-GNN) for predicting wall shear stress (WSS) distributions in coronary arteries under various stenosis configurations.

### Performance evaluation metrics

The evaluation encompasses a systematic investigation of stenosis geometries categorized by shape and position: concentric round, eccentric round, concentric oval, and eccentric oval stenoses. For concentric stenoses, severity is quantified by the stenotic area ratio (stenotic cross-sectional area divided by normal vessel area), where ratios of 10%, 30%, and 50% correspond to severe, moderate, and mild stenoses, respectively. For eccentric stenoses, the degree of eccentricity is characterized by the eccentric distance, defined as the displacement between the stenosis center and the vessel center, with values of 0.2 mm, 0.4 mm, and 0.6 mm representing increasing levels of geometric asymmetry. In addition, both single and double stenosis configurations are analyzed to evaluate model performance under anatomical complexities commonly encountered in clinical practice.

Model performance was assessed using a combined qualitative and quantitative evaluation framework. Qualitative evaluation employed three complementary approaches: (1) three-dimensional WSS distribution visualizations showing both CFD-calculated and model-predicted results for direct comparison; (2) flattened “unwrapped” surface heatmaps displaying WSS magnitude distributions across the arterial surface for detailed spatial analysis; and (3) absolute-error heatmaps illustrating pointwise differences between predicted and CFD-derived WSS values, enabling visual identification of systematic over- or underestimation patterns. Quantitative evaluation primarily relied on node-wise scatter plots together with the coefficient of determination (R^2^) and the Pearson correlation coefficient (R) to assess global agreement with CFD. In addition, Bland–Altman analysis was performed for representative stenosis cases to quantify systematic bias and 95% limits of agreement between predicted and reference WSS. To compare lesion-level hemodynamic signatures, axial mean WSS profiles along the centerline were computed from the three-dimensional fields and contrasted between each learning model and CFD. Classical error statistics including Mean Absolute Error (MAE), Median Absolute Error (Mdn AE), 75th percentile Absolute Error (75th AE), standard error of the prediction error, and Root Mean Squared Error (RMSE) are summarized in the corresponding tables to provide a complementary, multi-dimensional assessment of model accuracy.

### Performance in concentric coronary artery stenosis

In this section, we systematically evaluate and compare the predictive performance of three deep learning algorithms: MLP, U-Net, and PI-GNN, in concentric coronary artery stenosis scenarios. The analysis covers both round and oval geometries across three severity levels defined by the stenotic area ratio: mild stenosis (stenotic area ratio = 50%), moderate stenosis (stenotic area ratio = 30%), and severe stenosis (stenotic area ratio = 10%).

**Fig. 8:**
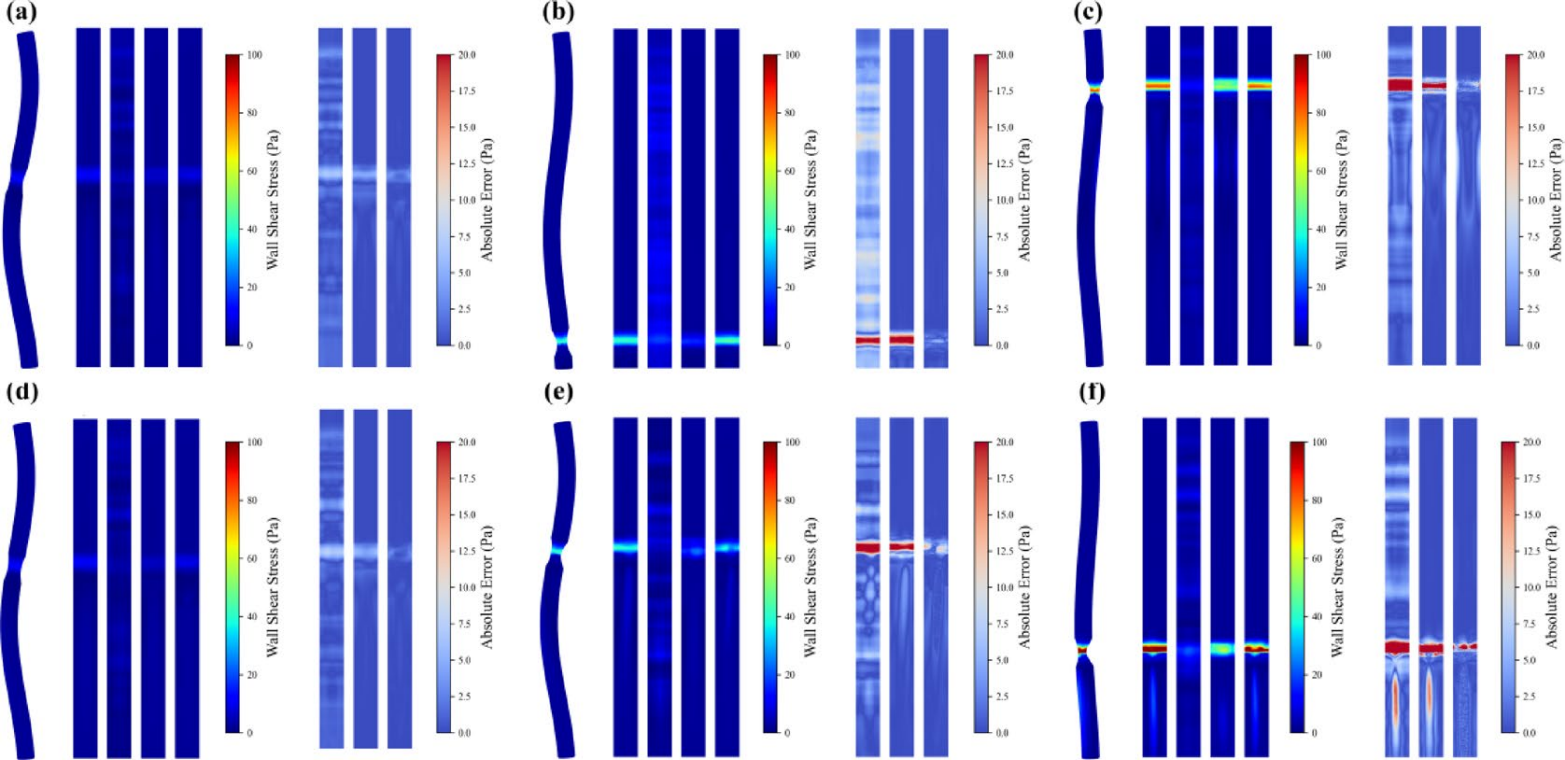
Comparisons between CFD results and ML predictions for concentric stenosis scenarios. In each subfigure, 3D WSS map (left) and the unwrapped surface plot (right) correspond to CFD, MLP, U-Net and PI-GNN, respectively.

Figure 8 presents CFD-calculated and model-predicted wall shear stress (WSS) distributions for these three severity levels. Specifically, Fig. 8a–c correspond to mild, moderate and severe concentric round stenoses, whereas Fig. 8d–f show the corresponding concentric oval stenoses, each with a single stenotic site. As shown in Fig. 8a–c, concentric round stenoses exhibit relatively uniform and symmetric WSS patterns owing to their axisymmetric geometry, and the stenotic area ratio directly modulates local WSS magnitude through flow acceleration. In the mild case (stenotic area ratio = 50%, Fig. 8a), the limited area reduction yields modest velocity increases, with peak WSS values around 10 Pa. As stenosis severity progresses to the moderate case (area ratio = 30%, Fig. 8b), the stronger area reduction substantially accelerates flow and elevates maximum WSS to approximately 40 Pa. Across these severities, PI-GNN consistently provides the closest match to CFD, accurately reproducing both the spatial extent and magnitude of WSS with small residual errors. U-Net correctly identifies the location of the stenotic region but systematically underestimates peak WSS, especially in the moderate and severe cases. By contrast, MLP predictions exhibit marked discrepancies, tending to overestimate WSS in nominally normal segments and underestimate critical WSS peaks at the stenotic throat. In the severe stenosis scenario (stenotic area ratio = 10%, Fig. 8c), where the pronounced area reduction produces WSS peaks on the order of 100 Pa, PI-GNN still maintains high accuracy with prediction errors of only a few pascals, whereas U-Net continues to underestimate maxima and MLP fails to capture the essential stenotic flow features.

In contrast, Fig. 8d–f depict concentric oval stenosis scenarios, which demonstrate distinct hemodynamic characteristics compared to round configurations. The elliptical cross-section creates preferential flow compression along the minor axis, typically inducing higher WSS values than equivalent area-ratio round stenoses. Moreover, oval stenoses frequently generate complex downstream flow patterns, including vortex formations and wake regions with elevated WSS values that may influence vascular remodeling and dilation risks. Across all severity levels, PI-GNN maintains superior performance, accurately capturing both the stenotic site WSS distributions and the downstream flow disturbances characteristic of oval geometries. U-Net predictions remain adequate but continue to underestimate WSS magnitudes, particularly in regions experiencing complex secondary flows. MLP performance deteriorates further in oval configurations, struggling with the geometric complexity and failing to accurately predict both stenotic and post-stenotic WSS patterns.

**Fig. 9:**
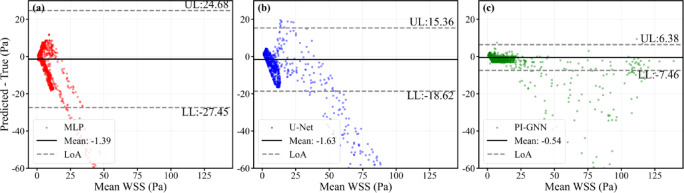
Node-wise Bland–Altman plots for severe concentric oval stenosis (stenotic area ratio = 10%). Upper limit (UL) and lower limit (LL) represent the average difference ± 1.96 standard deviations, which are calculated using all nodes.

Figure 9 presents node-wise Bland–Altman plots for the severe concentric oval stenosis case (stenotic area ratio = 10%), providing a complementary assessment of agreement between predicted and CFD-derived WSS. Although all three models exhibit small mean biases (MLP: − 1.39 Pa; U-Net: − 1.63 Pa; PI-GNN: − 0.54 Pa), their 95% limits of agreement differ substantially. PI-GNN achieves the tightest LoA (− 7.46 to 6.38 Pa), with most nodes clustered near zero difference and only minimal proportional bias across the full WSS range, indicating consistently small local errors. By contrast, MLP and U-Net show much wider LoA and a funnel-shaped pattern with increasing underestimation at higher WSS, confirming that only PI-GNN attains a level of agreement suitable for reliable identification of hemodynamically critical sites. The observed underestimation above approximately 25 Pa reflects an inherent imbalance in the training data, where extreme WSS peaks are confined to highly localized stenotic throats in the most severe cases while the vast majority of surface nodes exhibit moderate values (5–15 Pa), causing the MSE-based loss to disproportionately optimize for the dominant moderate-WSS range.

**Fig. 10:**
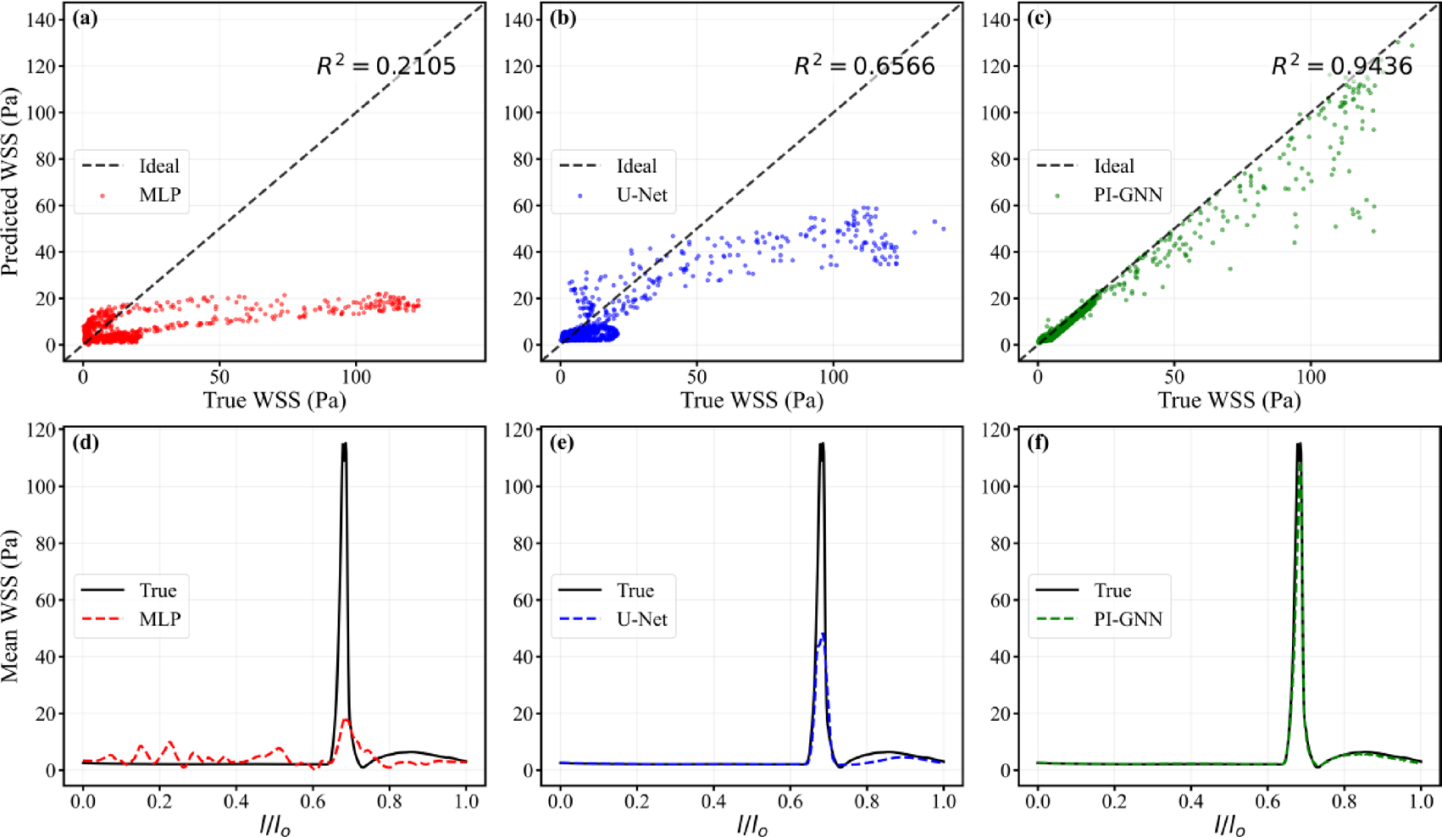
Node-wise scatter plots of predicted versus CFD-derived WSS (**a**–**c**) and axial mean WSS profiles along the normalized centerline $l/l_{0}$ (**d**–**f**) for MLP, U-Net, and PI-GNN in the severe concentric oval stenosis case (stenotic area ratio = 10%).

Figure 10 highlights the performance gap among the three architectures for this challenging lesion. The scatter plots confirm that PI-GNN achieves the strongest linear agreement with CFD (R^2^ = 0.9436), whereas U-Net attains only moderate correlation (R^2^ = 0.6566) and MLP performs poorly (R^2^ = 0.2105), with large dispersion around the identity line. The axial profiles provide a more mechanistic view of these differences: MLP fails to reproduce both the magnitude and location of the stenosis-induced WSS peak, resulting in a substantially flattened curve. U-Net captures the peak location but markedly underestimates its amplitude and oversmooths the downstream recovery region. In contrast, PI-GNN closely overlays the reference curve across the entire vessel, accurately resolving the sharp WSS elevation at the stenotic throat and the subsequent decay, thereby demonstrating its ability to preserve both global correlation and local hemodynamic structure.

**Fig. 11:**
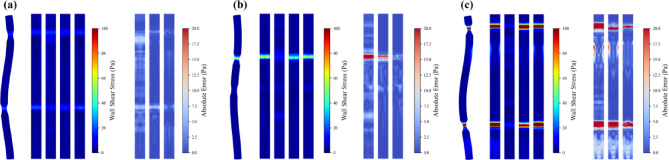
Comparisons between CFD results and ML predictions for dual concentric stenosis scenarios. In each subfigure, 3D WSS map (left) and the unwrapped surface plot (right) correspond to CFD, MLP, U-Net and PI-GNN, respectively.

We further evaluate model performance in more complex anatomical scenarios involving two concentric stenoses, as illustrated in Fig. 11. These configurations represent clinically relevant multi-vessel disease patterns where hemodynamic interactions between sequential stenoses create additional computational challenges.

Figure 11a illustrates two concentric round stenoses with mild severity (stenotic area ratio = 50% for both lesions). The presence of dual stenoses enlarges the region of abnormally elevated WSS, and the downstream stenosis exhibits slightly higher values than the upstream one owing to flow conditioning by the first lesion. MLP roughly identifies the stenotic locations but predicts blurred lesion boundaries and inaccurate peak values. U-Net effectively localizes both stenoses and delineates the downstream wake region, although it systematically underestimates peak WSS. PI-GNN provides the closest agreement with CFD, accurately capturing the WSS magnitude differences between the two stenoses with minimal residual error. Figure 11b shows dual concentric oval stenoses with asymmetric severity: a moderate upstream lesion (stenotic area ratio = 30%) followed by a mild downstream lesion (stenotic area ratio = 50%). This severity gradient generates pronounced WSS differences between the two sites, and the oval geometry further amplifies these effects through preferential flow compression. Under these more challenging conditions, MLP fails to reliably localize the stenotic regions. Both U-Net and PI-GNN correctly identify lesion locations and reproduce the severity-dependent WSS elevation, with PI-GNN demonstrating superior quantitative agreement with CFD. Figure 11c presents a mixed-geometry configuration with an upstream oval stenosis followed by a downstream round stenosis, both at severe level (stenotic area ratio = 10%). The elliptical stenosis produces higher WSS values than the circular one despite the identical area ratios, confirming the stronger flow compression associated with oval geometries. In this case, MLP predictions are largely unreliable, whereas PI-GNN markedly outperforms U-Net by accurately reproducing geometry-dependent WSS differences and capturing the complex downstream wake patterns.

**Fig. 12:**
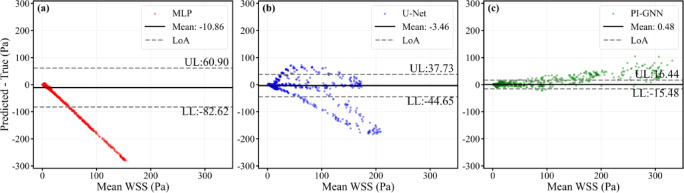
Node-wise Bland–Altman plots for dual concentric stenosis scenarios. Upper limit (UL) and lower limit (LL) represent the average difference ± 1.96 standard deviations, which are calculated using all nodes.

Figure 12 presents node-wise Bland–Altman plots for the dual concentric stenoses with mixed geometry, providing an agreement analysis between predicted and CFD-derived WSS under a more complex hemodynamic configuration. All three models exhibit non-zero mean biases (MLP: − 10.86 Pa; U-Net: − 3.46 Pa; PI-GNN: 0.48 Pa), but their 95% limits of agreement again differ markedly. PI-GNN attains the narrowest LoA (− 15.48 to 16.44 Pa) and a cloud of points concentrated around zero difference, indicating relatively small local errors and only mild proportional bias across the full WSS range. In contrast, MLP shows the widest LoA (− 82.62 to 60.90 Pa) with a pronounced downward trend, reflecting severe underestimation at higher WSS levels, while U-Net moderately reduces the bias yet still displays substantial scatter and systematic underprediction in high-WSS regions. Notably, PI-GNN exhibits a slight positive mean bias (+ 0.48 Pa) with mild overestimation in the high-WSS range, in contrast to the underestimation observed in the single-stenosis case (Fig. 9). We hypothesize that the graph message-passing mechanism accumulates upstream flow disturbance information that amplifies predictions at downstream stenotic sites, while the physics-inspired continuity constraint encourages the model to sustain elevated WSS throughout the inter-stenotic region rather than allowing partial recovery. These findings demonstrate that, even in the more demanding dual-stenosis scenario, only PI-GNN maintains a level of agreement with CFD that is compatible with reliable characterization of local hemodynamic extremes.

**Fig. 13:**
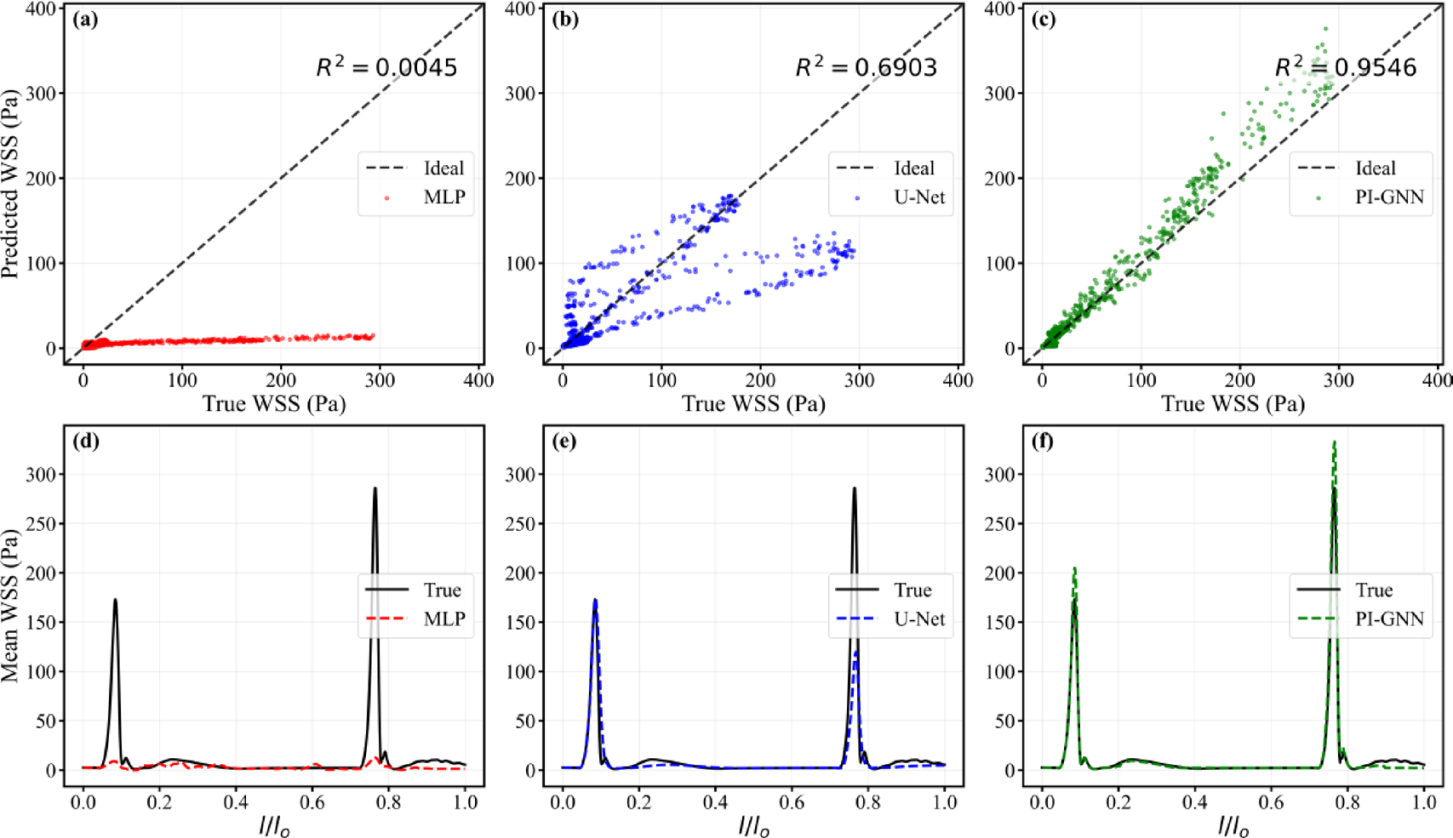
Node-wise scatter plots of predicted versus CFD-derived WSS (**a**–**c**) and axial mean WSS profiles along the normalized centerline $l/l_{0}$ (**d**–**f**) for MLP, U-Net, and PI-GNN in the dual concentric stenoses with mixed geometry.

Figure 13 compares model performance in the dual concentric stenoses with mixed geometry. The scatter plots show that PI-GNN achieves the strongest linear agreement with CFD (R^2^ = 0.9546), whereas U-Net provides only moderate correlation (R^2^ = 0.6903) and MLP essentially fails to recover the mapping (R^2^ = 0.0045), with predictions compressed into a low-WSS band. Consistently, the axial mean WSS profiles reveal that MLP almost completely flattens the two stenosis-induced peaks, and U-Net captures their locations but substantially underestimates their amplitudes. In contrast, PI-GNN closely overlaps the CFD reference along the entire centerline, accurately reproducing both peak magnitude and position, thereby preserving the key hemodynamic features of this complex lesion.

The quantitative performance metrics for concentric stenosis scenarios (Table 1) reveal clear performance differences among the three architectures. PI-GNN achieves the best overall accuracy, with the lowest error metrics (MAE = 0.81, Mdn AE = 0.15, 75th AE = 0.11, Std Error = 3.29, RMSE = 3.29) and the highest Pearson correlation coefficient (R = 0.98), indicating excellent agreement with CFD-predicted WSS distributions across concentric stenosis geometries and severity levels. U-Net shows intermediate performance with reasonable accuracy (MAE = 1.17, RMSE = 6.77, R = 0.91), effectively identifying stenotic regions but with a tendency to underestimate peak WSS values, particularly in severe stenosis cases where flow acceleration is most pronounced. MLP consistently underperforms, exhibiting substantially larger errors (MAE = 3.72, RMSE = 14.70) and poor correlation (R = 0.48), which is consistent with the qualitative observation that dimensionality reduction through dual autoencoders leads to loss of critical spatial information required to resolve sharp WSS gradients. Overall, the superior performance of PI-GNN supports the advantage of incorporating physics-informed constraints into the network architecture when modelling complex hemodynamics in concentric stenosis scenarios.

**Table. 1:** Predictive performance comparison of three deep learning models for WSS prediction in concentric coronary artery stenosis scenarios.

Model	MAE	Mdn AE	75th AE	Std Error	RMSE	Pearson R
MLP	3.72	1.54	2.44	14.69	14.70	0.48
U-Net	1.17	0.12	0.19	6.76	6.77	0.91
PI-GNN	0.81	0.15	0.11	3.29	3.29	0.98

### Performance in eccentric coronary artery stenosis

This section evaluates the predictive performance of the three deep learning algorithms (MLP, U-Net and PI-GNN) for eccentric coronary artery stenosis, which represents more complex geometric configurations commonly encountered in clinical practice. The analysis covers both round and oval stenosis shapes across three eccentric distances (0.2 mm, 0.4 mm and 0.6 mm), representing increasing levels of geometric asymmetry relative to the vessel centerline. All cases employ severe stenosis (stenotic area ratio = 10%) to examine model performance under the most challenging flow conditions, where both geometric eccentricity and strong flow acceleration strongly influence WSS.

**Fig. 14:**
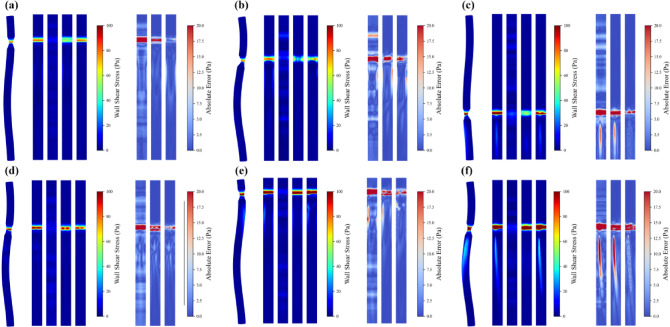
Comparisons between CFD results and ML predictions for eccentric stenosis scenarios. In each subfigure, 3D WSS map (left) and the unwrapped surface plot (right) correspond to CFD, MLP, U-Net and PI-GNN, respectively.

Figure 14 illustrates CFD-calculated and model-predicted WSS distributions for the three eccentricity levels. Figure 14a–c correspond to eccentric round stenoses, whereas Fig. 14d–f represent eccentric oval stenoses, each featuring a single lesion with progressively increasing eccentric distance. For eccentric round stenoses, geometric asymmetry has a marked impact on flow patterns. At the smallest eccentricity (0.2 mm, Fig. 14a), the WSS distribution remains broadly similar to the concentric case, with peak values around 80 Pa. As the eccentric distance increases to 0.4 and 0.6 mm in Fig. 14b, c, asymmetric flow acceleration produces sharper WSS peaks approaching 100 Pa, together with intensified downstream wake formation and vortical structures. These changes arise from the shifted flow constriction, which creates preferential flow channels and enhanced wall interaction on the stenotic side. Across all eccentricity levels, PI-GNN exhibits the closest agreement with CFD, accurately capturing both the stenotic WSS pattern and the downstream disturbances with small residual errors. U-Net reliably identifies the stenotic region but tends to underestimate peak WSS and to miss part of the wake structure, limiting its ability to characterize complex post-stenotic flow. MLP shows substantial inaccuracies, overestimating WSS in nominally normal segments while underestimating critical values at the stenotic throat, indicating difficulty in handling geometric asymmetry.

The eccentric oval stenosis scenarios in Fig. 14d–f constitute the most demanding test cases, combining elliptical cross-sectional compression with centerline displacement. These configurations generate WSS levels that exceed those of eccentric round stenoses with the same area ratio and produce pronounced downstream wake regions that are potentially relevant to vascular remodeling risk. The resulting geometric complexity induces large WSS fluctuations along the arterial surface, providing a stringent test of the prediction algorithms. PI-GNN again maintains excellent performance, closely reproducing the stenotic WSS distribution and successfully identifying abnormal downstream flow regions that are critical for clinical risk assessment. U-Net retains moderate capability in lesion localization but consistently underestimates peak WSS and fails to fully resolve the wake patterns. MLP performance degrades further in these cases, with poor localization of stenotic regions and large prediction errors throughout the arterial domain.

**Fig. 15:**
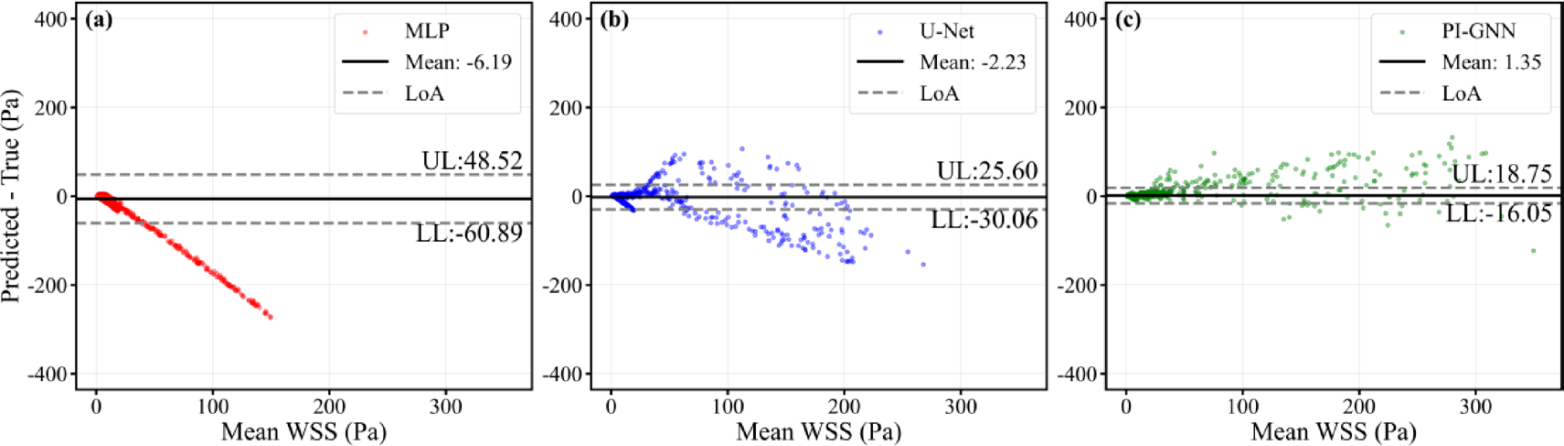
Node-wise Bland–Altman plots for severe eccentric oval stenosis (eccentric distance = 0.6 mm). Upper limit (UL) and lower limit (LL) represent the average difference ± 1.96 standard deviations, which are calculated using all nodes.

Figure 15 shows node-wise Bland–Altman plots for the severe eccentric oval stenosis case (eccentric distance = 0.6 mm). All three models exhibit relatively small mean biases (MLP: − 6.19 Pa; U-Net: − 2.23 Pa; PI-GNN: 1.35 Pa), but their limits of agreement differ substantially. PI-GNN yields the narrowest LoA (− 16.05 to 18.75 Pa) and a compact cloud of points around zero, indicating relatively small local errors and limited proportional bias.

**Fig. 16:**
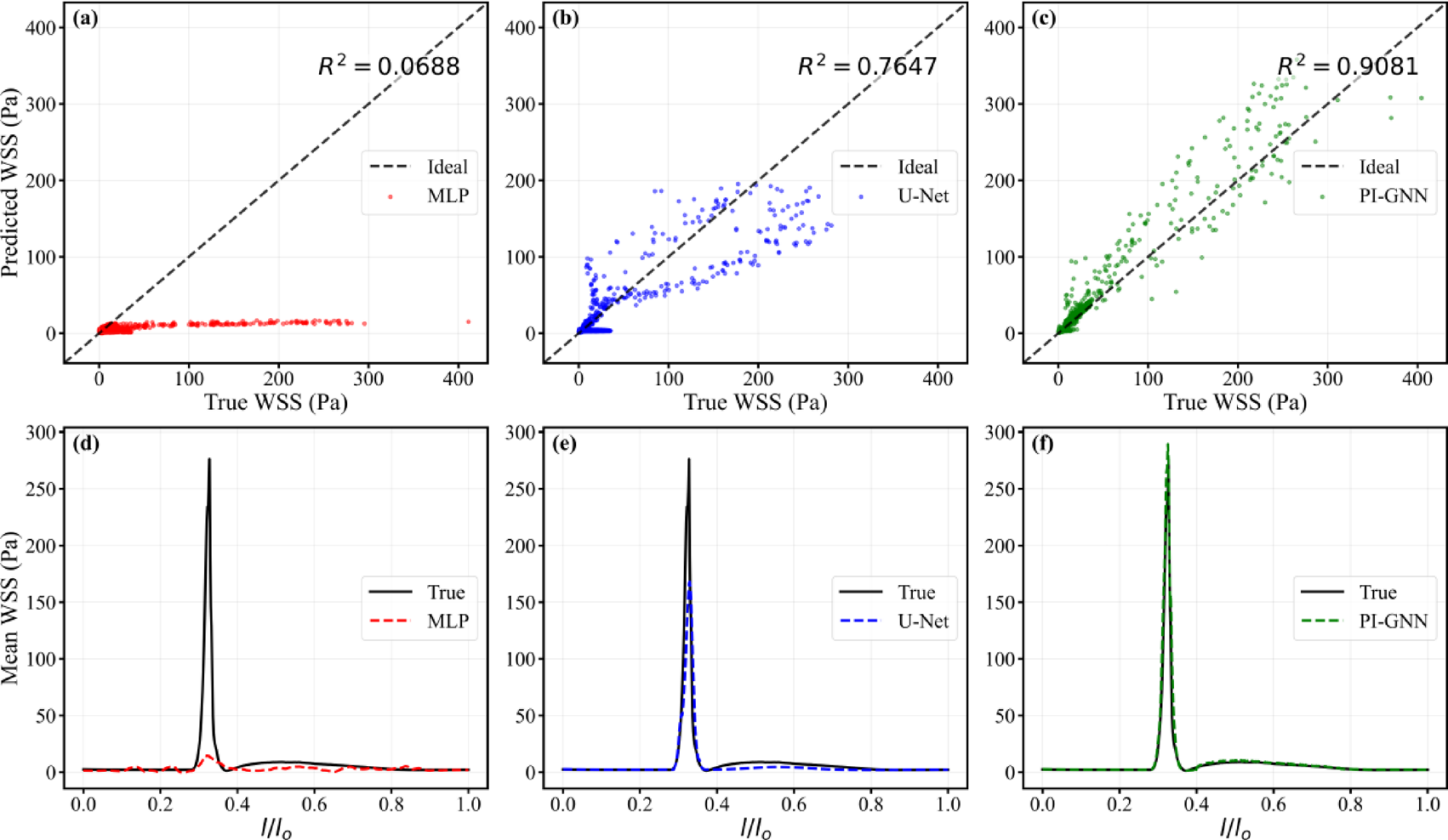
Node-wise scatter plots of predicted versus CFD-derived WSS (**a**–**c**) and axial mean WSS profiles along the normalized centerline $l/l_{0}$ (**d**–**f**) for MLP, U-Net, and PI-GNN in the severe eccentric oval stenosis (eccentric distance = 0.6 mm).

Figure 16 summarizes the corresponding correlation and axial WSS behaviour. PI-GNN attains the highest node-wise correlation with CFD (R^2^ = 0.9081), exceeding U-Net (R^2^ = 0.7647) and MLP (R^2^ = 0.0688). Consistently, the axial mean WSS profiles show that MLP markedly flattens the eccentric peak and underestimates WSS throughout the vessel, whereas U-Net captures the peak location but still underestimates its amplitude. PI-GNN closely follows the CFD reference curve, accurately reproducing both the height and position of the eccentric WSS peak, thereby preserving the key hemodynamic features of this lesion.

**Fig. 17:**
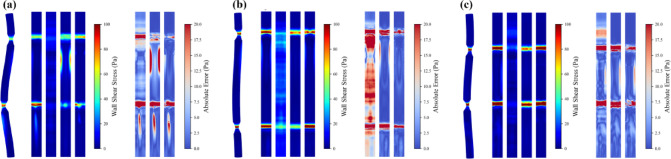
Comparisons between CFD results and ML predictions for dual eccentric stenosis scenarios. In each subfigure, 3D WSS map (left) and the unwrapped surface plot (right) correspond to CFD, MLP, U-Net and PI-GNN, respectively.

We further analyze model performance in dual eccentric stenosis configurations, as illustrated in Fig. 17. These scenarios represent the most complex geometric arrangements, combining multiple stenotic sites with centerline displacement effects. Figure 17a presents two eccentric round stenoses with severe stenosis (stenotic area ratio = 10%). The dual eccentric configuration expands abnormal WSS regions significantly, with both stenoses generating pronounced downstream wake patterns. MLP roughly identifies stenotic locations but lacks precision in boundary predictions. U-Net successfully detects both stenoses but exhibits mixed wake region performance: overestimating WSS in the first wake while underestimating values in the second wake. PI-GNN achieves superior performance, precisely localizing both stenotic regions and accurately capturing the first downstream wake pattern. Figure 17b depicts dual eccentric oval stenoses, representing the highest geometric complexity. Under these challenging conditions, MLP completely fails to localize stenotic regions effectively. Both U-Net and PI-GNN successfully identify stenotic locations, with PI-GNN demonstrating consistently superior accuracy and precisely capturing abnormal WSS distributions in the downstream wake region. Figure 17c presents a mixed-geometry scenario with upstream round and downstream oval eccentric stenoses, both at severe level. PI-GNN exhibits minimal prediction errors at both stenotic sites and successfully captures downstream wake patterns. The model accurately differentiates flow responses between different geometries, while U-Net shows reasonable localization but reduced wake prediction accuracy, and MLP fails to capture the geometric complexity.

**Fig. 18:**
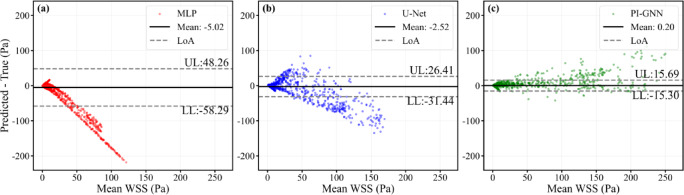
Node-wise Bland–Altman plots for dual eccentric stenoses with mixed geometry (eccentric distance = 0.6 mm). Upper limit (UL) and lower limit (LL) represent the average difference ± 1.96 standard deviations, which are calculated using all nodes.

Figure 18 presents node-wise Bland–Altman plots for the dual eccentric stenoses with mixed geometry (eccentric distance = 0.6 mm). All models show modest mean biases (MLP: − 5.02 Pa; U-Net: − 2.52 Pa; PI-GNN: 0.20 Pa), but the limits of agreement differ markedly. PI-GNN yields the narrowest LoA (− 15.30 to 15.69 Pa) with points densely clustered around zero, indicating relatively small local errors. U-Net provides intermediate agreement (LoA: − 37.44 to 26.41 Pa), while MLP shows the widest LoA (− 58.29 to 48.26 Pa) and pronounced underestimation at higher WSS levels.

**Fig. 19:**
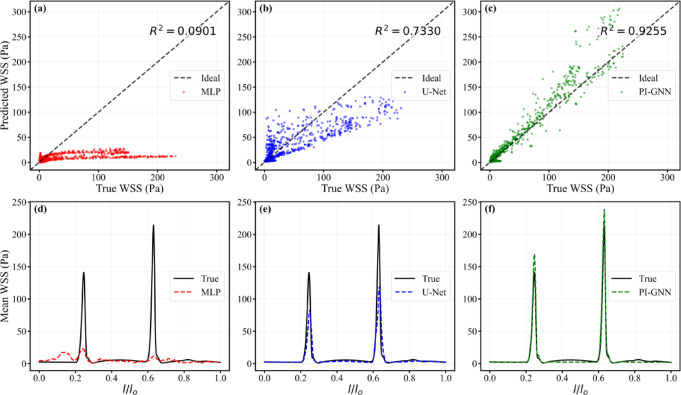
Node-wise scatter plots of predicted versus CFD-derived WSS (**a**–**c**) and axial mean WSS profiles along the normalized centerline $l/l_{0}$ (d–f) for MLP, U-Net, and PI-GNN in the dual eccentric stenoses with mixed geometry (eccentric distance = 0.6 mm).

Figure 19 confirms these trends from the perspective of correlation and axial WSS structure. PI-GNN attains the highest node-wise correlation with CFD (R^2^ = 0.9255), clearly outperforming U-Net (R^2^ = 0.7330) and MLP (R^2^ = 0.0901), whose predictions collapse into a narrow low-WSS band. In the axial mean WSS profiles, MLP severely flattens both eccentric peaks, and U-Net captures their locations but substantially underestimates their amplitudes. In contrast, PI-GNN closely overlaps the CFD reference along the centerline, accurately reproducing the magnitude and position of both WSS peaks and preserving the key hemodynamic features of the dual-lesion configuration.

The quantitative performance metrics for eccentric stenosis scenarios (Table. 2) reveal notable performance degradation across all models compared to concentric configurations, reflecting the increased complexity of asymmetric flow patterns. PI-GNN maintains the best predictive capability with MAE = 1.09, RMSE = 6.02, and Pearson correlation coefficient R = 0.90, demonstrating robust performance despite the geometric asymmetry and complex wake formation patterns characteristic of eccentric stenoses. U-Net shows moderate performance with MAE = 1.30, RMSE = 7.55, and R = 0.82, effectively capturing general WSS distributions but experiencing increased prediction errors in regions with complex secondary flows and asymmetric shear patterns typical of eccentric configurations. MLP exhibits substantially degraded performance with significantly higher errors (MAE = 5.57, RMSE = 12.23) and very poor correlation (R = 0.13), indicating its inability to handle the geometric complexity and spatial asymmetry inherent in eccentric stenoses. The performance ranking remains consistent with concentric scenarios (PI-GNN > U-Net > MLP), though all models show reduced accuracy due to the challenging nature of eccentric flow prediction. PI-GNN’s physics-informed constraints and graph-based geometric representation enable it to better capture the complex hemodynamic phenomena in eccentric stenoses, including flow separation, wake formation, and asymmetric WSS distributions that significantly challenge purely data-driven approaches.

**Table. 2:** Predictive performance comparison of three deep learning models for wall shear stress prediction in eccentric coronary artery stenosis scenarios.

Model	MAE	Mdn AE	75th AE	Std Error	RMSE	Pearson R
MLP	5.57	1.92	4.15	12.13	12.23	0.13
U-Net	1.30	0.13	0.70	7.53	7.55	0.82
PI_GNN	1.09	0.16	0.62	6.01	6.02	0.90

The comprehensive performance metrics across all stenosis configurations are summarized in Table. 3, representing the most challenging evaluation encompassing concentric and eccentric geometries, single and double stenoses, round and oval cross-sections, and complex mixed geometric combinations. PI-GNN consistently demonstrates superior predictive capability across all evaluated indicators, achieving the lowest error metrics: MAE = 1.05, Median AE = 0.15, 75th AE = 0.63, Standard Error = 5.63, and RMSE = 5.63. Most notably, PI-GNN attains the highest Pearson correlation coefficient (R = 0.94), indicating exceptional accuracy in capturing spatially complex WSS distributions across the entire spectrum of stenosis scenarios. U-Net shows moderate performance with reasonable accuracy (R = 0.85) and intermediate error levels (MAE = 1.39, RMSE = 7.62), effectively identifying stenotic regions and capturing general WSS patterns but systematically underestimating peak WSS values, particularly in complex configurations involving dual stenoses and eccentric geometries with mixed cross-sectional shapes. MLP consistently underperforms across all metrics, exhibiting substantially high errors (MAE = 4.39, RMSE = 14.6) and poor correlation (R = 0.24), with its inability to handle high-dimensional spatial relationships and complex geometric variations making it unsuitable for clinical WSS prediction applications. The superior performance of PI-GNN across this comprehensive evaluation demonstrates its physics-informed architecture’s effectiveness in incorporating fundamental fluid dynamics constraints to accurately model complex hemodynamic phenomena, maintaining consistent accuracy from simple single concentric stenoses to the most challenging dual eccentric configurations with mixed geometries.

**Table. 3:** Predictive performance comparison of three deep learning models for wall shear stress prediction in all coronary artery stenosis scenarios.

Model	MAE	Mdn AE	75th AE	Std Error	RMSE	Pearson R
MLP	4.39	1.84	4.01	14.58	14.6	0.24
U-Net	1.39	0.20	0.77	7.60	7.62	0.85
PI_GNN	1.05	0.15	0.63	5.63	5.63	0.94

This study was conducted on a Windows 10 workstation with AMD EPYC 9650 processors and 32 GB RAM. The CFD simulations were performed using ANSYS Fluent with computational time varying based on stenosis complexity, with individual models requiring 2–3 h to complete. To accelerate the dataset generation process, parallel computing was employed across multiple cores, reducing the total computational time for preparing the comprehensive dataset (i.e., CFD simulations of 1000 idealized coronary models across diverse stenosis configurations) to approximately 300 h. The machine learning models were implemented using TensorFlow 2.18 framework, with training accelerated by RTX 5070Ti GPU. Training duration varied significantly among architectures: the MLP framework required approximately 25 min for the dual autoencoder training process, U-Net training lasted around 3–4 h due to its end-to-end architecture complexity, while PI-GNN training extended to 4–5 h owing to its physics-informed constraints and curriculum learning strategy. The preparation of the comprehensive dataset required approximately 310 computational hours in total. However, once trained, all networks could generate complete WSS distributions within 1–2 s during inference, demonstrating the framework’s suitability for real-time clinical applications such as catheterization procedures and percutaneous coronary.

## Discussion

The results presented in Section “Results and discussion” demonstrate that PI-GNN achieves superior predictive performance compared to the U-Net and MLP baselines across all stenosis configurations. However, several aspects of the study design and methodology warrant further discussion, including limitations of the current framework and directions for future improvement.

While the SSM-based augmentation strategy effectively expands the training dataset from 40 subjects to 1,000 synthetic geometries while preserving statistical distributions of centerline curvature and tortuosity, the synthetic models employ idealized stenotic profiles that do not capture pathological irregularities such as calcified nodules, lipid cores, or ulcerated surfaces, nor do they account for imaging artifacts inherent in clinical CCTA acquisitions or patient-specific branching asymmetry and vessel tapering. Furthermore, the handcrafted input features are tightly coupled to the idealized geometric assumptions of the synthetic dataset, and the current experiments cannot disentangle the contributions of feature design, graph architecture, and physics-inspired loss terms to the observed performance. Additionally, the current model was trained under rigid-wall assumptions with Newtonian flow and a limited range of boundary conditions; its performance in fully patient-specific 3D coronary trees with compliant walls, non-Newtonian rheology, and imaging-derived uncertainties remains to be established. Residual underestimation also persists at extreme WSS values, as evidenced by the Bland–Altman analyses, warranting caution when interpreting very high shear peaks in clinical settings. The comparison among the three architectures also reveals important design trade-offs. The MLP’s poor performance is primarily attributed to its node-independent processing that lacks spatial neighborhood information, a limitation that becomes increasingly severe with rising stenosis severity and sharpening WSS gradients, while its non-end-to-end training strategy may further compound information loss. U-Net partially mitigates this through convolutional spatial encoding on the structured 2D grid, but its fixed receptive field and rigid grid topology limit its ability to faithfully represent irregular vessel surfaces, resulting in systematic peak WSS underestimation in complex configurations. PI-GNN’s graph-based representation offers a more natural encoding of the vessel surface topology, enabling flexible neighborhood aggregation that better captures the local geometric variations critical for accurate WSS prediction.

Future work will focus on incorporating these complexities to enhance the clinical applicability of the framework. Planned extensions include noise injection during training to improve robustness against imaging uncertainties, realistic plaque morphology modeling to better represent clinical pathology, extension to bifurcated geometries with compliant vessel mechanics, incorporation of diverse hemodynamic boundary conditions, and systematic uncertainty quantification to provide confidence intervals for clinical decision-making. Ablation studies to isolate the individual contributions of graph topology, physics-informed loss components, and input feature engineering are also warranted to further optimize the architecture.

## Conclusion

This study establishes a physics-informed graph neural network (PI-GNN) as an efficient and accurate framework for real-time wall shear stress prediction in stenotic coronary arteries. Across systematically constructed stenosis morphologies—including concentric and eccentric lesions, round and oval cross-sections, and single and dual stenotic sites—PI-GNN consistently outperformed U-Net and MLP baselines. At the global level, it achieved the lowest prediction errors (MAE ≈ 1 Pa, RMSE ≈ 6 Pa) and the highest correlation with CFD ground truth (R ≈ 0.94), while faithfully preserving the overall WSS distribution along the vessel centerline. Beyond conventional error metrics, node-wise Bland–Altman and correlation analyses provided a more stringent assessment of agreement. PI-GNN exhibited the smallest mean biases (typically |bias|< 2 Pa) and the narrowest 95% limits of agreement, generally within ± 15–20 Pa even in severe and dual-lesion scenarios, whereas U-Net and especially MLP showed much wider limits and pronounced proportional bias with progressive underestimation at high WSS. The MLP’s poor performance is primarily attributed to its node-independent processing that lacks spatial neighborhood information, a limitation that becomes increasingly severe with rising stenosis severity and sharpening WSS gradients, while its non-end-to-end training strategy may further compound information loss. U-Net partially mitigates this through convolutional spatial encoding on the structured 2D grid, but its fixed receptive field and rigid grid topology limit its ability to faithfully represent irregular vessel surfaces, resulting in systematic peak WSS underestimation in complex configurations. Scatter plots and axial WSS profiles confirmed that PI-GNN not only maintained high linear correlation (R^2^ often > 0.90) but also accurately reproduced the magnitude and location of stenosis-induced WSS peaks and post-stenotic recovery, thereby preserving the key hemodynamic structures that are most relevant for atherosclerotic risk assessment. Coupled with an inference time on the order of seconds, this level of agreement highlights the potential of PI-GNN to serve as a practical surrogate for CFD in time-critical clinical and research settings.

## Data Availability

All data generated and analysed during this study are available in the Zenodo repository at 10.5281/zenodo.17366694. The shared datasets include idealized coronary artery models constructed via a shape-statistics-based synthesis pipeline and the corresponding centerline, surface mesh, geometric descriptor and boundary-condition data used for CFD simulations and machine-learning training. These derived datasets are released under an open-access license to facilitate reproducibility and further research. The clinical coronary CTA data used to inform the statistical shape model were obtained from a publicly available, fully anonymized dataset (Coronary Atlas project, Gharleghi et al.) and are not redistributed here; they can be accessed directly from the original repository in accordance with its data-use policy. The shared resources include the coronary artery models constructed via the shape-statistics-based synthesis pipeline, together with the corresponding centerline, surface mesh, geometric descriptor and boundary-condition data used for CFD simulations and machine-learning training. These derived datasets are released under an open-access license to facilitate reproducibility and further research. The clinical CCTA data used to inform the statistical shape model were obtained from the publicly available dataset reported by Gharleghi et al.^24^ and are not redistributed here; they can be accessed directly from the original repository in accordance with its data-use policy.
